# A syllable-character collaborative model for enhanced Pinyin and Chinese recognition

**DOI:** 10.1371/journal.pone.0325045

**Published:** 2025-07-07

**Authors:** Zeyuan Chen, Cheng Zhong, Danyang Chen

**Affiliations:** 1 School of Computer, Electronics and Information, Guangxi University, Nanning, Guangxi, China; 2 Key Laboratory of Parallel, Distributed and Intelligent Computing in Guangxi Universities and Colleges, Guangxi University, Nanning, Guangxi, China; Commonwealth Scientific and Industrial Research Organisation, AUSTRALIA

## Abstract

In Chinese speech recognition, end-to-end speech recognition models usually use Chinese characters as direct output and perform poorly compared with other language models. The main reason for this phenomenon is that the relationship between Chinese text and pronunciation is more complex. Inspired by the learning process of Chinese beginners, who first master initials, finals, and pinyin before learning characters, we propose the Syllable-Character Collaborative Model (SCCM), which incorporates these phonetic elements into the training process. Additionally, we design a Pinyin-Ensemble module that employs an ensemble learning approach to reduce pinyin recognition errors, which in turn leads to a reduction in text recognition errors. Experiments on AISHELL-1 show that our approach not only reduces pinyin and character error rates compared to a prior end-to-end method using pinyin as auxiliary information, but also achieves a 45.7% relative reduction in Character Error Rate (CER) over the AISHELL-1 baseline.

## Introduction

Automatic Speech Recognition (ASR) [1] is the process of converting human speech into corresponding text sequences. Most languages, such as those using Latin or Greek alphabets, are phonetic scripts, meaning their written characters relatively correspond to their pronunciation [2, 3]. However, Chinese is more closely tied to the meaning of the characters rather than their pronunciation [4]. This fundamental difference makes the mapping between speech and characters more complex, posing greater challenges for speech recognition in Chinese compared to other languages [5, 6].

Chinese uses pinyin as a phonetic notation system, where each character is transcribed into a syllable composed of an initial and a final. For example, the character “ ä¸­ ” (zhong) has the initial ‘zh’ and the final ‘ong’. Chinese language learners typically begin by mastering initials and finals, which they then combine into pinyin syllables to understand pronunciation rules and establish connections between pronunciation and Chinese characters.

In traditional speech recognition [7–9] systems, researchers use acoustic models to convert audio into corresponding phonemes through a series of independent modules. These phonemes are then combined with a pronunciation dictionary and language model to produce the most likely text sequence. Traditional ASR models have excellent capabilities in modeling language features and perform well across various languages. However, the independent optimization of disparate modules leads to complexity in system development, as it requires meticulous alignment of individual component objectives with the overall system goals, which is challenging and does not fully exploit the complementary strengths of the components.

In recent years, end-to-end speech recognition [10–12] has gained widespread attention and achieved great success across many languages. This approach simplifies the architecture of traditional systems by treating the target text as the direct output and learning the mapping from speech to text directly from paired data. However, compared to recognition tasks in other languages, the complex mapping relationship between Chinese speech and text, compounded by the challenges of homophones and polysemy recognition, often results in suboptimal performance for end-to-end systems.

To solve the above problems, we propose Syllable-Character Collaborative Model (SCCM). Our model includes a shared encoder and three decoders, which decode pinyin, initials and finals, and characters respectively. To mitigate the complexity of directly mapping speech to characters, we fuse the Pinyin embedding vector with the speech feature vector as the input of the character decoder. Pinyin embedding vectors share acoustic representations through the multi-task learning framework, which has been proven to reduce the impact of noise in speech [13, 14] and narrowing the search space. While speech features represent local acoustic states and are more focused on physical properties like pitch and duration, they are less effective at capturing contextual information. In contrast, pinyin embeddings provide a higher-level semantic analysis of speech, offering richer contextual information to the decoder. To ensure effective coordination among these modules, we designed a Pinyin-Ensemble (PE) module, which synthesizes the outputs of the three decoders to generate more accurate pinyin. The generated pinyin is further processed through the Pinyin Feature (PF) module to produce pinyin embedding vectors, which are used as inputs to the next round of text decoding. To achieve unified optimization across modules, we developed a joint loss function. Finally, to reduce errors caused by homophones, we incorporate Pycorrector before text output.

In this paper, we use a multi-task learning model that incorporates pinyin, initials and finals to improve the accuracy of speech recognition. Our main contributions are summarized as follows:

- We propose Syllable-Character Collaborative Model, which includes a shared encoder and multiple decoders. We adopt different decoding methods for different modeling units, and use the same encoder and joint loss for training to achieve coordination between modules, thus improving the final recognition accuracy.
- We design Pinyin-Ensemble module using the idea of ensemble learning, which integrates pinyin, initials and finals, and Chinese characters to produce more accurate predicted pinyin. Subsequently, we utilize Pinyin Feature module to transform the pinyin into embedding vectors, which are concatenated with the shared feature vectors and input into the next round of text decoder, significantly reducing the error rate of the text.
- Experimental results on the AISHELL-1 dataset demonstrate that our model achieves significant improvements over baseline models.

The remainder of this paper is organized as follows: In the Related Work section, we review previous studies. The framework architecture and methodology are detailed in the Methodology section. In the Experiment section, we present the experimental setup and analyze the results. Finally, the findings are concluded in the Conclusion section.

## Related work

### End-to-end speech recognition models

Speech recognition technology has evolved from the era of Gaussian Mixture Models and Hidden Markov Models [15] (GMM-HMM) to the Deep Neural Networks and Hidden Markov Models [16, 17] (DNN-HMM) era, eventually advancing to the current state-of-the-art end-to-end models. Currently, there are three main types of end-to-end technologies in the field of speech recognition:

Connectionist Temporal Classification [18–20] (CTC): CTC is commonly employed to handle sequences where the lengths of input and output are not aligned. A major drawback of CTC is its assumption that inputs at each time step are independent, which does not hold in speech recognition where there is semantic information across time steps. Recent work [21] mitigates this via BERT/GPT-2 [22, 23] knowledge transfer, but its effectiveness is limited due to the inherent mismatch between pre-trained text representations and acoustic features [24].

Attention-based Encoder-Decoder architectures [25–27] (AED): Most research [28–30] on ASR primarily utilizes AED based on transformers [31], because it can capture long-range semantic relationship. However, they suffer from high latency and error propagation in noisy conditions. The mixed method [32] alleviates this problem by jointly decoding pinyin and characters, but it requires additional fuzzy pinyin datasets.

Recurrent Neural Network Transducer [33–35] (RNN-T): While RNN-T is suitable for streaming ASR, it is inherently limited when applied to non-streaming, full-audio ASR tasks due to its left-to-right decoding nature and lack of access to future context [36]. And it is ill-suited for our multi-task framework [37] due to RNN-T lacks the flexibility to integrate external intermediate predictions into its decoding process. Prior studies [38] show that RNN-T’s shared joiner causes gradient conflicts when handling divergent tasks.

To address the alignment and long-range dependency issues in speech recognition, we use CTC and encoder-decoder architecture at the same time in this paper. CTC solves the problem of monotonic alignment between audio frames and sub phoneme units (such as pinyin and finals) by providing hard alignment, while the encoder decoder architecture can capture long-range dependencies and contextual information [39, 40]. Compared with RNN-T, CTC has better alignment ability in full audio tasks, can handle long sequences and avoid the limitations of streaming decoding, making it more suitable for our multitasking framework.

### Chinese modeling units

In traditional speech recognition, modeling units [41, 42] are the fundamental units used to represent the mapping between speech signals and text. In traditional Chinese ASR, modeling units often include phonemes, initials and finals, and Pinyin. However, in strict end-to-end ASR, Chinese characters or words are used directly as speech modeling units.

The choice of modeling units significantly impacts recognition performance. A comparative study [43] of acoustic modeling units of deep neural networks for large-vocabulary Chinese speech recognition has proved that combining multiple modeling units performs better than each individual modeling unit. Further research [44] has explored end-to-end Chinese speech recognition. This study examined modeling units at three scales: context-dependent phonemes, syllable tones, and Chinese characters. Phonemes lack direct correspondence to Chinese orthography, increasing grapheme-phoneme alignment complexity. Using word-based models in Chinese presents sparsity issues due to the large vocabulary.

Motivated by these findings, our approach integrates multiple modeling units into a unified framework. CTC decoding is applied to Pinyin and initials-finals because of its alignment-free training and efficiency in modeling sub-word units. The attention-based decoder is employed for Chinese characters, leveraging its strong capability in capturing long-range dependencies and contextual information.

## Methodology

**Fig 1:**
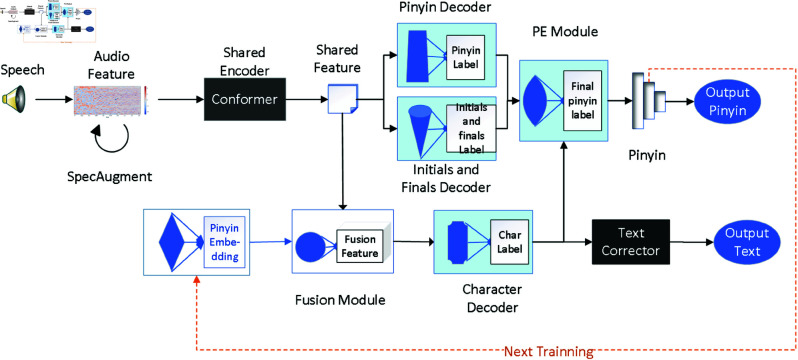
The structure of SCCM.

The structure of our SCCM is shown in Fig 1. The training of SCCM consists of three phases. In phase 1, the pinyin decoder and initials-finals decoder are trained independently using CTC loss, focusing on acoustic-phonetic alignment. These decoders predict the corresponding pinyin and initials-finals. In phase 2, the character decoder is introduced, and the system undergoes joint training with a multi-task loss function. During this phase, the outputs of all three decoders are integrated into the Pinyin-Ensemble (PE) module, which refines the pinyin predictions. Finally, in phase 3, the model undergoes full end-to-end training, with each round refining pinyin and text decoding through the iterative feedback of predictions. We use the predicted pinyin obtained from the previous round to generate the pinyin embedding vector through our pinyin feature module and fuse it with the shared feature as the input for the text decoder.

### Shared encoder module

In this paper, we employ the conformer [45] as the encoder module. It contains 6 conformer blocks, which are composed of four stacked components: the FeedForward Network module (FFN), the Multi-Head Self-Attention module (MHSA), the Convolutional module (Conv), and a second FeedForward Network module. By combining convolutional operations with self-attention mechanisms, conformer effectively captures both short-term and long-term features in speech signals. For an input $x=\left(x_{1},x_{2},x_{3},...x_{T}\right)$ to the Conformer module, where T is the number of time steps, the shared feature $h=\left(h_{1},h_{2},...h_{T}\right)$ can be expressed as:

h=Conformer(x) (1)

### Pinyin decoder module

In the pinyin decoding module, we use the CTC decoding module to obtain the probability distribution of the pinyin sequence, CTC technology allows us to bypass the need for alignment between audio and pinyin outputs and can handle input-output sequences of varying lengths. It has been demonstrated that using pinyin as modeling units achieves the highest accuracy when decoded with CTC [46]:

$$P_{pinyin}(y|h)=\prod_{t=1}^{T}p(y_{i}|h_{t}),y_{i}\in V_{pinyin}$$ (2)

where *P*_*pinyin*_(*y*|*h*) is the probability distribution of the pinyin sequence, $y=\left(y_{1},y_{2},y_{3},...,y_{u}\right)$ is the predicted target sequence, *u* is the length of the prediction sequence *y*, u≤T, *i* is the index of each target pinyin in prediction sequence *y*, $V_{pinyin}$ is the pinyin dictionary we create. And the n-best method [47] is used to select the best-performing pinyin sequences, which can enable us to obtain the global optimal solution:

$$y^{pin}=\underset{y\in B(V_{pinyin})}{argmax}P_{pinyin}(y|h)$$ (3)

where ${\text{y}}^{\text{pin}}$ is the best-performing pinyin sequences, $B(V_{pinyin})$ is the set of legal pinyin paths formed in the pinyin dictionary after removing spaces and merging consecutive repeated characters.

### Initials and finals decoder module

Similarly, we use the CTC decoding module to obtain the probability distribution of the sequence of initials and finals, and we use the n-best method to select initials and finals. The calculation formula is as follows:

$$P_{if}(y|h)=\prod_{t=1}^{T}p(y_{i}|h_{t}),y_{i}\in V_{if}$$ (4)

$$y^{if}=\underset{y\in B(V_{if})}{argmax}P_{if}(y|h)$$ (5)

where *P*_*if*_(*y*|*h*) is the probability distribution of the initials and finals sequence, $V_{if}$ is the initials and finals dictionary we create, *i* is the index of each target initial or final in prediction sequence *y*, $B(V_{if})$is the set of legal initials and finals paths formed in the initials and finals dictionary after removing spaces and merging consecutive repeated characters. The repetition prediction mechanism of CTC effectively handles multi-syllable words and connected speech phenomena in Chinese, thereby improving the robustness of initial and final recognition.

### Pinyin feature module and feature fusion module

**Fig 2:**
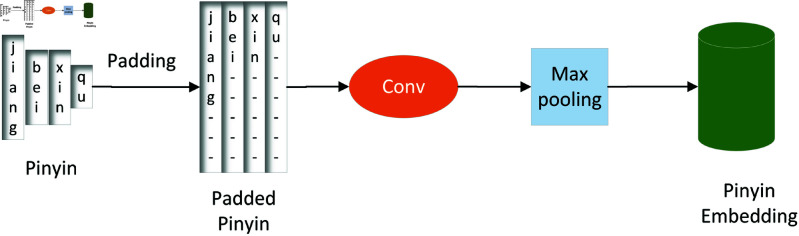
The pinyin feature module.

**Fig 3:**
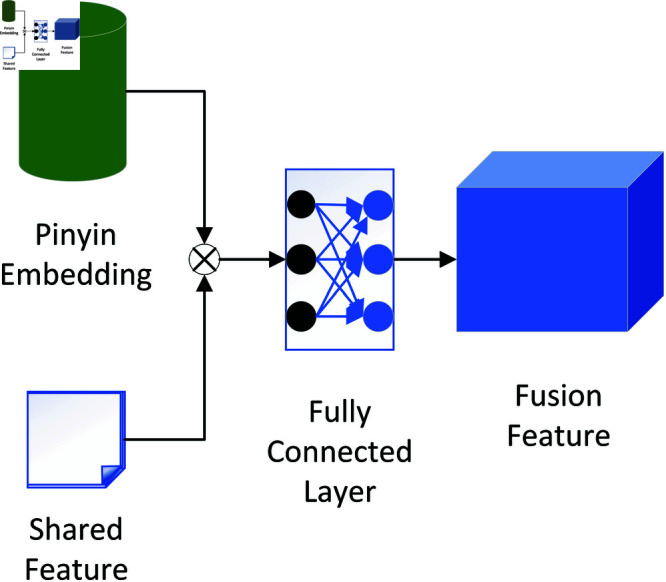
The feature fusion module. ⊗ denotes vector concatenation.

As a bridge between speech and text, Pinyin provides phonetic information that can aid character decoding by offering finer-grained pronunciation details [48, 49]. Inspired by ChineseBERT [50], which integrates pinyin and glyph information to enhance the representation of Chinese text, we also incorporate pinyin embeddings into our model. However, unlike ChineseBERT, which is designed for text-based tasks, our focus is on improving speech recognition. In our approach, we fuse the shared features with the pinyin features to help the character decoder better understand the context information of the input. A pinyin representation rarely exceeds 6 characters (for example, “zhuang" for “ å£(r) ”). However, to ensure consistency in the input sequence length across the model, we set the length of each pinyin vector to eight, with the remaining positions padded with the symbol ‘ - ’. To obtain a compact and informative Pinyin representation, we first apply a Convolutional Neural Network (CNN) to extract hierarchical phonetic features from the padded Pinyin sequence. This CNN-based Pinyin embedding captures sub-syllabic dependencies, allowing the model to better understand pronunciation variations. In order to adapt to the input of the text decoder, we concatenate the shared features with the pinyin embedding vector into a new vector and convert this new vector into the input vector of the character decoder through a fully connected layer. This fusion reinforces phonetic constraints during decoding, thereby mitigating homophone errors and improving text generation quality [51, 52]. The two modules are shown in Figs 2 and 3. The calculation formulas are as follows:

$$y_{i}^{pad}=Pad(y_{i-1}^{pp})$$ (6)

$$y_{i}^{emb}=Conv(y_{i}^{pad})$$ (7)

$$h_{i}^{conc}=Concatenate(h,y_{i}^{emb})$$ (8)

$$h_{i}^{char}=FC(h_{i}^{conc})$$ (9)

where $y_{i-1}^{pp}$ is the output of the ensemble learning module in the previous round of training, $y_{i}^{pad}$ is padded pinyin, $y_{i}^{emb}$ is our pinyin embedding feature vector, $h_{i}^{conc}$ is concatenated vector, $h_{i}^{char}$ is the output feature of feature fusion module, *i* refers to the index of our training rounds. *FC* is the fully connected calculation function.

### Character decoder module and Pycorrector

We use the Transformer as the character decoder. During the initial training round, the decoder input consists solely of the shared features generated by the encoder. The formula for calculation is as follows:

$$y_{0}^{char}=Transformer(h)$$ (10)

In subsequent rounds, the fusion features are used as inputs, with characters as outputs. The calculate detail is as follows:

$$y_{i}^{char}=Transformer(h^{char}),\;i=1,2,3...$$ (11)

where ${\text{y}}^{\text{char}}$ is the output of decoder, *i* refers to the index of our training rounds. Additionally, at the end of the process, we incorporate a Chinese text correction tool called Pycorrector to refine the decoder’s output.

$$y^{fc}=Pycorrector(y^{char})$$ (12)

where ${\text{y}}^{\text{fc}}$ is our final Chinese text. This tool analyzes text using a combination of contextual and linguistic rules, along with statistical and deep learning models, to identify potential errors and suggest appropriate corrections. Pycorrector can effectively detect common spelling, grammatical, and phonetic errors in Chinese text. Its integration into our workflow enhances the accuracy of text post-processing, thereby improving the overall performance of the speech recognition model.

### Pinyin-ensemble module

Since the accuracy of pinyin affects the accuracy of the final text, improving pinyin correctness is a critical issue. We pre-trained Pinyin-Ensemble (PE) module, which is constructed with the following ten base classifiers: Logistic Regression [53], Support Vector Classifier (SVC) [54], K-Nearest Neighbors (KNN) [55], Decision Tree [56], Random Forest [57], Gradient Boosting [58], Naive Bayes [59], Multi-layer Perceptron (MLP) [60], AdaBoost [61], and Bagging Classifier [62]. Each base classifier is trained individually on the extracted character-level features from pinyin, syllable, and Chinese character data. A Random Forest classifier acts as the meta-learner, receiving predictions from each base classifier as input features. Data set is split with an 80-20 ratio for training and testing, using a 5-fold cross-validation approach. Hyperparameters were optimized for each base classifier based on grid search results. The calculation formula is as follows:

$$y^{pp}=Jc(y^{pin},y^{if},y^{char})$$ (13)

where the inputs include pinyin, initials and finals, and Chinese pinyin, and the output ${\text{y}}^{\text{pp}}$ is the predicted pinyin of this round of training. *Jc* is the calculation function of our Pinyin-Ensemble module. The main purpose of this module is to integrate the pinyin information obtained from these three decoding methods, resulting in more accurate pinyin and reducing the impact of subsequent pinyin errors on text decoding.

### Training loss

In order to achieve unified optimization between modules, we optimize weight sum of the loss functions of different modules uniformly. The calculation formula is as follows:

$$L_{combine}=0.25*L_{CTC}^{pinyin}+0.25*L_{CTC}^{if}+0.5*L_{CE}^{char}$$ (14)

where *L*_*combine*_ is the combined loss, $L_{CTC}^{pinyin}$ and $L_{CTC}^{if}$ is the CTC loss of the pinyin and initials and finals, and $L_{CE}^{char}$ is the cross-entropy loss of the character.

## Experiment

### Data set and metrics

In this paper, we use the AISHELL-1 dataset [63] in 16 kHz WAV format to verify the performance of all the models. AISHELL-1 contains high-quality Mandarin speech recorded from multiple channels including iOS, Android, and microphones, thus covering diverse acoustic conditions. Each speech is represented as 80-dimensional filterbank coefficients computed every 10ms with a 25ms window length. All feature sequences are normalized using the mean and variance of each audio sample. SpecAugment [64] is employed to augment audio data for model training.

For both pinyin and text, we adopt Character Error Rate (CER) [65, 66] as the final evaluation metric, which is the most recognized metric in speech recognition. The calculation formula of CER is as follows:

$$CER=\frac{S+D+I}{N}$$ (15)

where *S* is the number of substitutions, *D* is the number of deletions, *I* is the number of insertions and *N* is the number of characters.

### Implementation details

The proposed model consists of six encoder layers, and six character decoder layers. The dimensions used in the model are as follows: the model dimension is 512, the embedding dimension is 512, and both the key and value dimensions of conformer are 64. We use the Adam optimizer with a learning rate of 0.001, following the same optimizer settings as the transformer model. To further prevent overfitting, label smoothing and dropout are applied, both with a probability of 0.1. All models are trained for 60 epochs with a batch size of 32.

### Experimental result and analysis

In Table 1, we compare the results of our model SCCM on AISHELL-1 dataset with commonly used ASR models, including: AISHELL-1 Baseline [63], SpeechTransformer [67], Conformer, CNN with CTC, Wav2vec2.0 [68], Dual-Decoder [32]. All models are evaluated on both Pinyin and Character levels, enabling a comprehensive assessment across phonetic and textual representations. Our proposed model consistently outperforms all baselines, achieving the lowest CER (6.4%) and pinyin CER (3.1%). In contrast, the Dual-Decoder model attains competitive results but relies on an additional fuzzy Pinyin dataset, introducing extra data dependency. Our method, trained solely on standard AISHELL-1 data, demonstrates superior performance without such reliance. The simultaneous reduction in both character and pinyin error rates indicates a systematic improvement rather than a result of random variation. We use CTC for initials and finals and pinyin, which can prove that CTC is more suitable for these than transformer.

**Table 1:** CER of our model and commonly used ASR models on AISHELL-1 test sets.

Models	CER	
	Pinyin	Text
*Baseline*	6.15	11.97
*CNNwithCTC*	4.96	10.21
*SpeechTransformer*	5.03	10.47
*Conformer*	3.20	7.30
*wav*2*vec*2.0	2.80	7.20
*DUAL*–*DECODER*	2.65	6.60
*SCCM*	2.41	6.50

In order to explore the contribution of phonetic information and tone information of our performance, we designed an ablation experiment whose results are shown in Table 2. Dc refers to using a text decoder, Dp means using a pinyin decoder, Dif refers to using an initials and finals decoder, PE/PE (Dp & Dif) means combining the outputs of decoders to generate new pinyin. Fusion means the pinyin embedding vectors will be fed into the text decoder. ‘✓’ indicates that the module is included.

**Table 2:** Ablation study of our model on AISHELL-1 test sets.

Modules	*Dc*	*Dp*	*Dif*	*Fusion*	PE(Dp&Dif)	*PE*	CER	
							Pinyin	Text
	✓						—–	8.20
	✓	✓		✓			3.54	7.83
**SCCM**	✓		✓	✓			3.69	7.92
	✓	✓	✓	✓	✓		2.55	6.73
	✓	✓	✓	✓		✓	2.41	6.50

We observe that using only the character decoder does not yield satisfactory results. Incorporating the pinyin decoder greatly reduces the character error rate, which indicates that pinyin directly corresponds to the pronunciation of speech and can provide more phonetic information. By comparing the second and third rows, we find that pinyin is more effective than initials and finals in assisting character decoding and results in a lower CER. Furthermore, by integrating both pinyin and initials/finals information through our Pinyin-Ensemble (PE) module, we achieve a 28.0% reduction in pinyin error rate (from 3.54% to 2.55%) and a 14.0% reduction in text CER (from 7.83% to 6.73%). This improvement confirms that combining multiple syllable unit information through ensemble learning can significantly reduce error rates.

To explore how the Pycorrector contributes to the performance of our model, we conduct an ablation experiment, whose result is shown in Table 3. Although the improvement might seem not significant, it indicates that Pycorrector effectively reduces errors in the predicted text. To further demonstrate the effectiveness of Pycorrector, we show some examples in Table 4. Case 0 and case 1 show the state that SCCM with and without Pycorrector are both correct. Pycorrector can help us solve some personal names problems as in case 2. In case 3, we find that it may make mistakes when matching some uncommon words. We can see the final output text of SCCM without Pycorrector may still contain a small number of errors due to misdecoding of pinyin or syllables as in case 4, Pycorrector further optimizes the character-level output and improves accuracy by correcting the generated text.

**Table 3:** Ablation study of pycorrector on AISHELL-1 test sets.

Models	CER	
	Pinyin	Text
SCCMWithoutPycorrector	2.45	6.53
*SCCM*	2.41	6.50

**Table 4:** Example output of SCCM with and without Pycorrector.

Case	SCCM without Pycorrector	SCCM	Ground Truth
0	苹果此举是为了节约用电量	苹果此举是为了节约用电量	苹果此举是为了节约用电量
1	博士	博士	博士
2	陈延希穿着粉色上衣	陈妍希穿着粉色上衣	陈妍希穿着粉色上衣
3	黄蓉穿着它不仅刀剑不入	黄蓉穿着它不仅刀枪不入	黄蓉穿着它不仅刀剑不入
4	完善主地承包经营全流市市场	完善土地承包经营权流转市场	完善土地承包经营权流转市场

## Conclusion

In this paper, we propose the Syllable-Character Collaborative Model. The model fully utilizes the units of Chinese speech and simultaneously performs decoding tasks for initials and finals, pinyin and Chinese characters. We also design a Pinyin-Ensemble module to integrate the outputs of these three tasks for learning, improving the recognition accuracy of pinyin and text. At the same time, we add Pycorrector to reduce the homonym errors. The results on the test set of AISHELL-1 dataset show that the proposed model outperforms commonly used mainstream ASR models on AISHELL-1 dataset.
